# Therapeutic potential of the metabolic modulator phenformin in targeting the stem cell compartment in melanoma

**DOI:** 10.18632/oncotarget.14321

**Published:** 2016-12-28

**Authors:** Tiziana Petrachi, Alessandra Romagnani, Adriana Albini, Caterina Longo, Giuseppe Argenziano, Giulia Grisendi, Massimo Dominici, Alessia Ciarrocchi, Katiuscia Dallaglio

**Affiliations:** ^1^ Laboratory of Translational Research, Department of Scientific Direction, Arcispedale S. Maria Nuova-IRCCS, Reggio Emilia, Italy; ^2^ Scientific and Technologic Park, IRCCS MultiMedica, Milan, Italy; ^3^ Skin Cancer Unit, IRCCS-Arcispedale Santa Maria Nuova, Reggio Emilia, Italy; ^4^ Dermatology Unit, Second University of Naples, Naples, Italy; ^5^ Department of Medical and Surgical Sciences for Children & Adults, University Hospital of Modena and Reggio Emilia, Modena, Italy

**Keywords:** melanoma, cancer stem cells, phenformin, therapy, ALDH

## Abstract

Melanoma is the most dangerous and treatment-resistant skin cancer. Tumor resistance and recurrence are due to the persistence in the patient of aggressive cells with stem cell features, the cancer stem cells (CSC). Recent evidences have shown that CSC display a distinct metabolic profile as compared to tumor bulk population: a promising anti-tumor strategy is therefore to target specific metabolic pathways driving CSC behavior. Biguanides (metformin and phenformin) are anti-diabetic drugs able to perturb cellular metabolism and displaying anti-cancer activity. However, their ability to target the CSC compartment in melanoma is not known. Here we show that phenformin, but not metformin, strongly reduces melanoma cell viability, growth and invasion in both 2D and 3D (spheroids) models. While phenformin decreases melanoma CSC markers expression and the levels of the pro-survival factor MITF, MITF overexpression fails to prevent phenformin effects. Phenformin significantly reduces cell viability in melanoma by targeting both CSC (ALDH^high^) and non-CSC cells and by significantly reducing the number of viable cells in ALDH^high^ and ALDH^low^-derived spheroids. Consistently, phenformin reduces melanoma cell viability and growth independently from SOX2 levels. Our results show that phenformin is able to affect both CSC and non-CSC melanoma cell viability and growth and suggests its potential use as anti-cancer therapy in melanoma.

## INTRODUCTION

Melanoma is the most aggressive, highly metastasizing and therapy-resistant skin tumor, with increasing incidence in the last decades. B-RAF inhibitors (B-RAFi) and the recently approved immune-checkpoint inhibitors ipilimumab and nivolumab/pembrolizumab have improved the progression-free survival of metastatic melanoma patients, however a large number of patients do not respond and many display disease relapse [1–3]. In the last few years, the contribution of cancer stem cells (CSC) to drug resistance has been clarified in many cancer types, including breast cancer and leukemia [4–6]. Conventional therapies kill mainly regular tumor cells while sparing CSC, leading to drug resistance and therapeutic failure. However, in melanoma, CSC existence and the selectivity/specificity of markers used for CSC isolation have been highly questioned in the last few years [7]. Evidence suggests that melanoma tumor growth is orchestrated by subpopulations of tumor-maintaining cells that can dynamically switch from a more differentiated state, and vice versa [8]. Nevertheless, many report also support the hierarchical CSC model in melanoma and several melanoma CSC markers have been identified so far, including the multi-drug resistance ABCB5 transporter and aldehyde dehydrogenase (ALDH) enzymes [9, 10]. We and others have recently shown the ability of ALDH activity to select for melanoma CSC [10–12]. High ALDH levels protect melanoma cells from apoptosis while ALDH1 blockade prevents tumor relapse [10–13].

It has become widely accepted that alterations of tumor cell metabolism are hallmarks of cancer [14, 15] and that targeting tumor cell metabolism is a promising anti-cancer strategy [16]. CSC seem to have specific metabolic profiles [17, 18], suggesting that targeting regulators of cancer cell metabolism might be a valuable CSC-eradicating approach. Biguanides, among which metformin and phenformin are the most widely known, are organic compounds with hypoglycaemic properties. Metformin is the first medication used to treat type II diabetes worldwide. Interestingly, biguanides display anti-cancer properties. Epidemiological evidences indicate a 30% lower risk for diabetic patients under metformin treatment to develop cancer [19, 20]. Our group has recently confirmed the anti-tumor effect of metformin and phenformin in breast cancer by targeting both tumor and microenvironment cells [21–23]. Biguanides act mainly by inhibiting mitochondrial oxidative phosphorylation (OXPHOS) through the blockade of mitochondrial complex-1 [24]. In melanoma cells, OXPHOS plays an important role in ATP production [25] and recent preclinical studies targeting melanoma cell mitochondrial bioenergetic metabolism have shown to be effective [26–29]. However, the effect of biguanides on melanoma growth and invasion is controversial. On one side, metformin seems to inhibit melanoma development [30, 31] and prevent invasion and metastasis [32]. On the other side, metformin accelerates B-RAF mutated melanomas growth *in vivo* by sustaining angiogenesis [33]. Different reports have shown the ability of metformin to selectively kill cancer stem cells [34, 35] also by reverting their quiescent state [36]. As a consequence, the combination of metformin with chemotherapy targeting the non-stem like compartment of the tumor is promising [37]. Recent findings suggest that other biguanides affect melanoma cell growth [38], possibly by reducing stem cell features [39]. Among these, phenformin strongly reduces melanoma growth and when combined with the B-RAFi PLX4720 gives a significant therapeutic advantage. Although phenformin seems to target specifically slow cycling melanoma cells [40], the direct effect on the CSC compartment of this tumor is unknown.

In the present work, we investigated the ability of phenformin to target the CSC compartment in melanoma by analyzing primary and metastatic melanoma cells both in monolayer cell cultures and 3D spheroids. We show that phenformin, but not metformin, abrogates melanoma cell growth and invasion in 2D and 3D models and affects both CSC and non-CSC cells in melanoma.

## RESULTS

### Phenformin decreases melanoma cell viability in both monolayer and spheroids cell cultures

**Figure 1:**
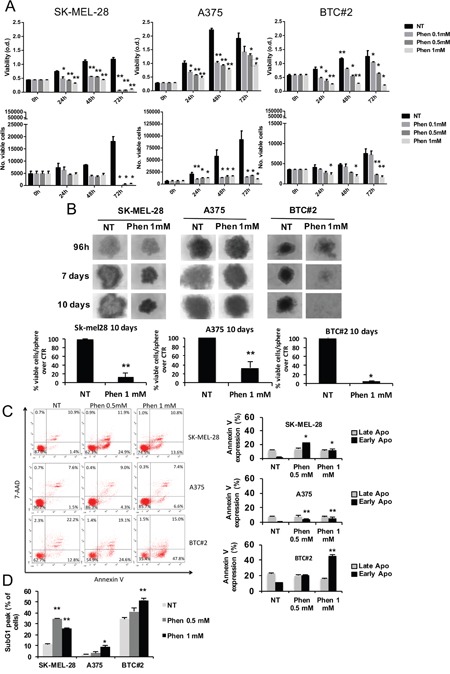
Phenformin reduces melanoma cell viability in both 2D and 3D models **A**. Melanoma cells were seeded, treated with 0.1-1mM phenformin and MTT assay (upper panel) or blue trypan cell counting (lower panel) were performed up to 72h after treatment. **B**. Melanoma cells were seeded in ultralow-attachment plates in complete medium for 96h. Once formed, spheroids were treated with 1mM phenformin and photographed at indicated timepoints (upper panel). At day 10, spheroids were harvested, mechanically disaggregated and viable cells were counted by trypan blue staining. Data represent the mean ±SD of the experiment performed in triplicate and are represented as the % of viable cells/spheroid over untreated (NT) spheroids. Student T-test was performed for statistical analysis of data (* p<0.05; ** p<0.01). **C**. SK-MEL-28, A375 and BTC#2 cells were treated with the indicated concentrations of phenformin for 72h, then subjected to Annexin V/7-AAD staining. The % of cells positive for both Annexin V and 7-AAD represent cells in late apoptotic phase, while cells annexin-V-positive and 7-AAD-negative are in early stages of apoptosis. **D**. Cells were treated as above, then subjected to flow cytometric propidium iodide staining. The % of cells in the SubG1 peak, which is indicative of apoptosis, is shown. Results are representative of three independent experiments and are shown as mean ± SD. Student T-test was performed for statistical analysis of data by comparing NT with phen-treated cells; * p<0.05.

First, we tested biguanides toxicity on melanoma cells. Besides SK-MEL-28 and A375 cells, we included the primary melanoma cell line BTC#2 in the analysis as a representative specimen of B-RAF-mutated melanoma cells established from a primary aggressive melanoma [41]. In accordance with previous findings [37], phenformin reduced melanoma cell viability by MTT (Figure 1A, upper panel) and cell proliferation by trypan blue cell counting starting from 24h after stimulus up to 72h (Figure 1A, lower panel). Interestingly, although biguanides interfere with cell metabolism, we observed similar results between MTT, a mitochondrial metabolism-sensitive viability assay, and trypan blue cell counting analyses. Since cell responses in 3D-cell cultures are similar to *in vivo* behavior [42], we also tested the effect of phenformin on melanoma spheroids by measuring cell viability by trypan blue cell counting 10 days after treatment. First of all, we observed a slight, but not significant, decrease in the number of viable cells/sphere over time in untreated SK-MEL-28 and BTC#2 spheroids (data not shown). This putatively reflects the different sensitivity of these cells to the microenvironmental conditions generated in the spheroid subcompartments, such as suboptimal nutrition and low oxygen supply [43]. When melanoma-derived spheroids were treated with phenformin, we observed a strong reduction in SK-MEL-28 and BTC#2 sphere size and morphology (Figure 1B, upper panel) as well as the number of viable cells in all cell lines upon treatment (Figure 1B, lower panel). Contrarily, the size and shape of A375-derived spheroids was only slightly affected by the treatment (Figure 1B). In line with the decrease in cell viability observed in monolayer cell cultures upon treatment with phenformin, we noticed a stronger effect of the drug on BTC#2-derived spheroids as compared to the other melanoma cell lines (Figure 1B). Interestingly, treatment of melanoma spheroids with a lower dose of phenformin (0.5mM) for 10 days was still able to reduce melanoma sphere-size (SK-MEL-28 and BTC#2) and the number of viable cells/sphere (Supplementary Figure 1A and 1B).

As opposite to what observed for phenformin, no considerable effect of metformin on cell viability and proliferation was observed in monolayer cell cultures (Supplementary Figure 2A). In melanoma-derived spheroids, the number of viable cells/sphere at day10 was significantly reduced by metformin in BTC#2 and SK-MEL-28-derived spheroids, but not in A375 ones (Supplementary Figure 2B). Although the effect of phenformin and metformin in spheroids was similar in some cell lines (e.g. SK-MEL-28, Supplementary Figure 1B and Supplementary Figure 2B), all together our data suggest that phenformin is more effective than metformin in reducing melanoma cell viability.

To characterize phenformin effect on melanoma cell death, cells were treated with 0.5 and 1 mM phenformin for 72h, then harvested for Annexin V/7-AAD or PI staining. We observed a strong and significant induction of early-phase apoptosis by phenformin in all cell lines. This effect was dose-dependent for A375 and BTC#2 cells, while phenformin 0.5mM showed a stronger effect than 1mM in SK-MEL-28 cells (Figure 1C). These results were confirmed by PI-staining at the same timepoint (Figure 1D) and indicate that phenformin induces apoptosis in melanoma cells.

### Phenformin, but not metformin, abrogates melanoma cell invasion in 3D spheroid models

**Figure 2:**
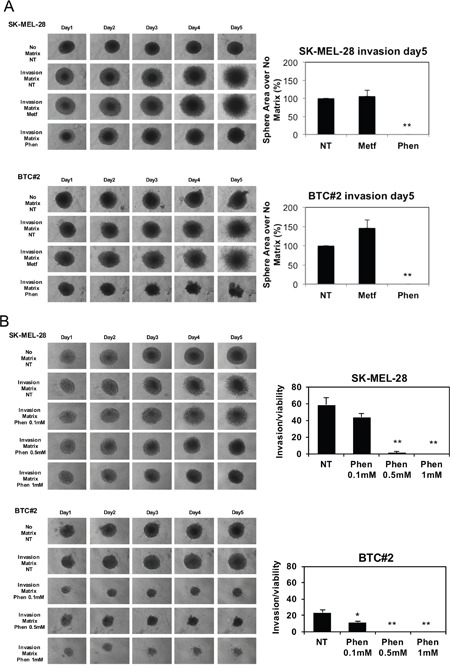
Phenformin, but not metformin, reduces melanoma spheroids invasion **A**. Melanoma cells were resuspended in spheroid formation ECM, seeded in a 3D culture 96-well plate and maintained up to 72 h. Then, invasion matrix was added and after gel formation, complete medium containing either vehicle (NT) or 10mM metformin or 1mM phenformin was added. A set of spheroids was maintained in absence of invasion matrix (no matrix). Invasion area at day5 was calculated by using ImageJ software. Bar graphs display the area of invasion of NT or treated spheroids over no matrix and are expressed as a % over NT. **B**. Spheroids were obtained as in A. Then, 0.1-1mM Phen was added to each well and pictures were taken at each timepoint. At day5, invasion matrix was digested, spheroids were disaggregated and viable cells were counted by trypan blue cell count. Bar graphs display the ratio between invasion (area of invasion in NT and treated spheroids over no matrix) and cell viability (viable cells in NT and treated spheroids over no matrix). Error bars represent mean ±SD of three independent experiments. Student T-test was performed for statistical analysis of data (* p<0.05; ** p<0.01).

*In vivo*, phenformin inhibits B-RAF-mutated melanoma cell growth [40], however its ability to reduce or inhibit melanoma metastatic process has not yet been assessed. To determine if phenformin affects melanoma cell metastasis, we performed a 3D-spheroid cell invasion assay on melanoma-spheroids in presence or absence of biguanides to evaluate the cell motility. Metformin failed to reduce, and even promoted, cell invasion both in SK-MEL-28 and BTC#2 derived spheroids (Figure 2A). By contrast, phenformin completely blocked melanoma cell invasion in both cell types (Figure 2A). Since phenformin affects melanoma cell viability and growth (Figure 1A and 1B), we analyzed invasion as well as cell viability in the same spheroid by enzymatic digestion of the physical bonds between the tumor cells and the extracellular matrix. This procedure allowed the quantification of cell viability in spheroids by trypan blue dye exclusion. As shown in Figure 2B, the ability of phenformin to decrease melanoma spheroids invasion is independent from phenformin-induced decrease in cell viability. Moreover, lower doses of phenformin (0.5 mM) were still able to significantly decrease cell invasion both in SK-MEL-28 and BTC#2 cells (Figure 2B). Similar results were observed when spheroids were pre-treated with phenformin for 72h and allowed to invade in absence of the drug, indicating that phenformin fails to select for invasive cells in melanoma spheroids. In these conditions, metformin did not impair melanoma cell invasion (Supplementary Figure 3). All together these data demonstrate that phenformin decreases melanoma cell invasion in 3D models.

### MITF overexpression is not protective against phenformin inhibitory effects

**Figure 3:**
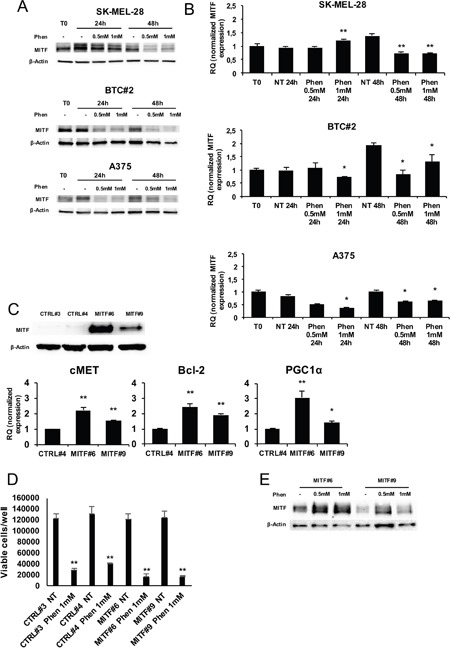
MITF is not protective against phenformin in melanoma cells **A**. Total cell lysates of melanoma cells treated with or without 0.5-1mM phenformin up to 48h were immune-blotted with anti-human MITF antibody. **B**. mRNA was extracted from the same cells as in A and MITF expression by real-time PCR was assessed. **C**. Two MITF overexpressing-A375 clones (MITF#6 and 9) and two control clones (CTRL#3 and 4) were lysed either for MITF expression by western blotting (upper panel) or for cMET, Bcl-2 and PGC1α mRNA analysis by Real Time PCR (lower panel). **D**. MITF#6, #9, CTRL#3, #4 cell clones were seeded and treated with 0.5-1mM phenformin. Trypan blue cell count at 72h was performed and the number of viable cells/well is shown. Data are the mean ±SD of the experiment performed in triplicate. Student T-test was performed for statistical analysis of data (* p<0.05; ** p<0.01). **E**. Total cell lysates of MITF#6 and #9 clones treated with or without 0.5-1mM phenformin for 48h were harvested and immunoblotted for MITF expression. Western blottings are representative of an experiment performed in triplicate (three biological replicates). β-actin was used as loading control.

In the attempt to define the molecular basis of phenformin inhibitory effect on melanoma cells, we tested whether it could affect the expression of MITF (Microphthalmia-associated transcription factor) that, besides being a melanocytic lineage-specific marker, it is also a pro-survival factor in melanoma [44, 45]. Indeed, phenformin induced a remarkable reduction of MITF protein and mRNA levels in all cell lines by western blotting and qRT-PCR (Figure 3A and 3B). To evaluate whether forced expression of MITF could protect from phenformin cytotoxic effects, we constitutively over-expressed MITF in melanoma cells; A375 cells, expressing a very low basal level of MITF [46], were transfected with a MITF-expressing vector or with an empty vector and single clones were generated by antibiotic selection. Two clones expressing different levels of MITF were selected: MITF#6 (high expression level) and MITF#9 (medium expression level) (Figure 3C). We used CTRL#3 and CTRL#4 clones as controls. First we verified that the MITF overexpressed protein was functional in our system. We analyzed the expression of well-established MITF target genes (Bcl-2, cMET and PGC1-α) in MITF clones by real time PCR. As shown in Figure 3C (lower panel) MITF overexpression increases the expression of the three genes in a MITF-dose dependent manner, thus confirming that MITF is functional in transfected cells. However, we failed to observe any difference in cell viability after treatment with phenformin between CTRL and MITF-overexpressing cells (Figure 3D), indicating that MITF is not sufficient to rescue phenformin cytotoxic activity in melanoma cells. No effect on MITF exogenous protein stability was observed upon treatment (Figure 3E).

### Phenformin decreases stem cell traits in melanoma

**Figure 4:**
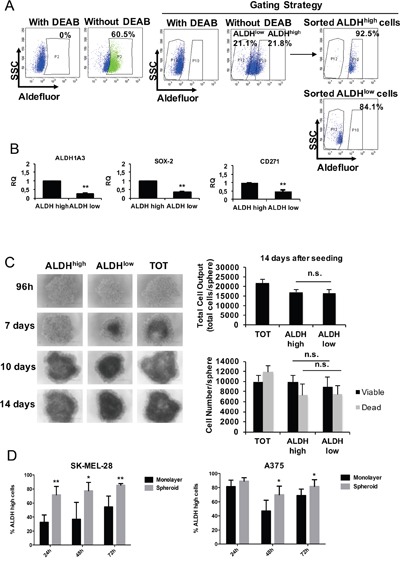
ALDHhigh melanoma cells behavior in 2D vs 3D cell culture models **A**. Representative Aldefluor analysis in SK-MEL-28 cells. Control cells incubated with Aldefluor inhibitor (DEAB) were used to identify ALDH^high^ and ALDH^low^ cells. Purity of sorted populations was checked. **B**. Just sorted ALDH^high^, ALDH^low^ and bulk (total, TOT) cells were immediately lysed for mRNA analysis by real-time PCR for the expression of CSC markers. Data are the mean ±SD of the experiment performed in triplicate. Student T-test was performed for statistical analysis of data (* p<0.05; ** p<0.01). **C**. Sorted ALDH^high^ and ALDH^low^ cells were seeded in complete medium in order to form spheroids and photographed at different timepoints. At day 14, spheroids were mechanically disaggregated and viable cells were counted by trypan blue. Bar graphs display total cell outputs (viable+dead cells) per spheroid (right, upper graph) or the cell number/sphere (right, lower graph). **D**. A375 and SK-MEL-28 cells were seeded at the same density in normal (monolayer) and ultralow-attach (for spheroids) plates in complete medium. Cells were harvested up to 72h and analyzed with Aldefluor by FACS analysis. The % of ALDH^high^cells at the different timepoints in the two cell culture conditions is shown. Error bars represent mean ±SD of five independent experiments. Student T-test was performed for statistical analysis of data (* p<0.05; ** p<0.01; n.s. not significative).

We have recently shown that ALDH^high^ cells from melanoma specimens and cell lines retain CSC features and are highly resistant to conventional therapies [10]. Therefore, we asked whether phenformin is able to target CSC in melanoma. To this aim, we isolated ALDH^high^ and ALDH^low^ melanoma cells by fluorescence-activated cell sorting as previously shown [10]. We chose to sort from SK-MEL-28 and A375 cells since only 0.8 % of BTC#2 cells are ALDH^high^ (Supplementary Figure 4A). As expected, ALDH^high^ melanoma cells express significantly higher levels of ALDH1A3, SOX2 and CD271 than ALDH^low^ cells by real time PCR (Figure 4B), western blotting (SOX2/ALDH1A1, Supplementary Figure 4B) and FACS analysis (CD271, Supplementary Figure 4C). ALDH^low^ cells slightly overexpressed MITF, consistent with its role in determining the differentiation towards the melanocyte lineage; as opposite, MITF main regulator, SOX10, was equally expressed in the two populations (Supplementary Figure 4D). Next, we evaluated the behavior of sorted ALDH^high^ and ALDH^low^ SK-MEL-28 (Figure 4) and A375 cells (Supplementary Figure 5) in 2D and 3D models. Sorted ALDH^high^ and ALDH^low^ populations were seeded on ultra-low attachment plates to form spheroids, then monitored up to 14 days post-seeding. Both ALDH^high^, ALDH^low^ and bulk (unsorted) cells were able to form spheroids in these conditions, yet ALDH^high^ cells generated slightly larger spheroids as compared to ALDH^low^ ones (Figure 4C and Supplementary Figure 5A, left panels). At day 14, total cell output of ALDH^high^-derived spheroids was similar to that of ALDH^low^ ones (Figure 4C and Supplementary Figure 5A, right panels). In accordance, the number of viable cells was not significantly different in ALDH^high^ derived spheroids as compared to ALDH^low^ derived ones, as shown by trypan blue cell counting (Figure 4C and Supplementary Figure 5A, right panels). When melanoma cells grown as monolayer were compared with spheroids for ALDH activity, we observed a significant increase in the number of ALDH^high^ cells in 3D cell cultures as compared to monolayer cell cultures. This suggests that melanoma cells grown as spheroids may increase the CSC compartment by upregulating ALDH activity (Figure 4D), however further experiments are needed to clarify this point. We also observed a strong variation of ALDH^high^ cell number in melanoma 2D cell cultures over time (Figure 4D). Although we don't have a full explanation on these results, it is possible that ALDH levels fluctuate in culture also in response to progressive metabolic changes that take place during the different phases of the cell cycle and as a function of cell density [47, 48].

**Figure 5:**
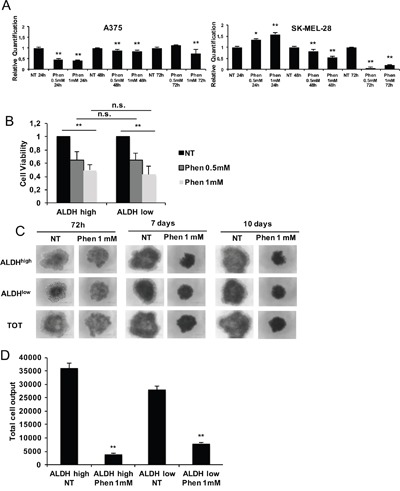
Phenformin targets both ALDHhigh (CSC) and ALDHlow (non-CSC) melanoma cells **A**. mRNA was extracted from melanoma cells treated with or without 0.5-1mM phenformin up to 72h. Then, ALDH1A isoforms expression by real-time PCR was assessed. We chose to measure ALDH1A3 and ALDH1A1 levels for A375 and SK-MEL-28 cells respectively as they are the most expressed ALDH isoforms in each cell type (data not shown). **B**. Sorted ALDH^high^ and ALDH^low^ SK-MEL-28 cells were seeded in complete medium on 96-wells plates and treated with 0.5-1 mM phenformin. Cell viability at 48h was measured by trypan blue cell count. **C**. Sorted ALDH^high^ and ALDH^low^ SK-MEL-28 cells were seeded in complete medium on ultralow-attach plates and treated with 1mM phenformin up to 10 days. Photographs of treated spheroids were taken at different timepoints. **D**. At day 10, spheroids were mechanically disaggregated and viable and dead cells were counted by trypan blue. Bar graphs display total cell outputs (viable+dead cells) per spheroid. Error bars represent mean ±SD of three independent experiments. Student T-test was performed for statistical analysis of data (* p<0.05; ** p<0.01).

**Figure 6:**
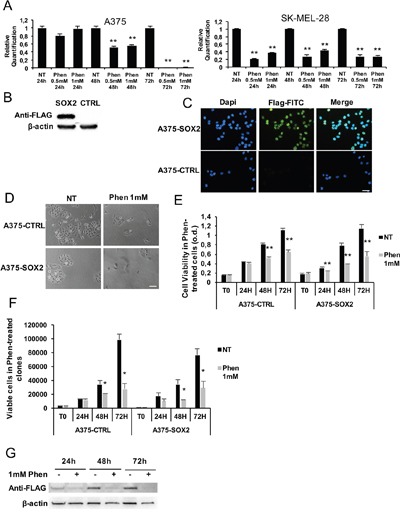
SOX2 overexpression in melanoma is not protective against phenformin **A**. SOX2 expression was assessed by real-time PCR in A375 and SK-MEL-28 cells treated with or without 0.5-1mM phenformin up to 72h. **B**. SOX2 overexpressing-A375 clone and a control clone were lysed for SOX2 expression by western blotting using an anti-flag antibody. **C**. Immunofluorescence was performed on A375-SOX2 clones by using anti-flag antibody (green) and dapi for nuclear staining (blue). The selected clone contains 100% flag-positive cells (scale bar: 25μm). **D**. Representative pictures of SOX2 and CTRL clones treated with or without 1mM phenformin at 72h (scale bar: 25μm). **E**. A375-CTRL and -SOX2 cell clones were seeded and treated with 0.5-1mM phenformin. MTT assay up to 72h was performed and cell viability is shown at each timepoint (o.d. optical density; ** p<0.01). **F**. Trypan blue cell count up to 72h was performed on the same cells as in D. The number of viable cells is shown at each timepoint. Data are the mean ±SD of the experiment performed in triplicate. Student T-test was performed for statistical analysis of data (* p<0.05). **G**. Total cell lysates of A375-SOX2 clone treated with or without 1mM phenformin were harvested up to 72h and immune-blotted for SOX2 expression using an anti-flag antibody. Western blottings are representative of an experiment performed in triplicate. β-actin was used as loading control.

In order to assess phenformin effects on the CSC compartment in melanoma, we isolated ALDH^high^ and ALDH^low^ melanoma cells by fluorescence activated cell sorting and analyzed them in 2D and 3D models in presence or absence of phenformin. In monolayer cell cultures, phenformin reduced ALDH isoforms expression in melanoma cells (Figure 5A) and ALDH^high^ and ALDH^low^ melanoma cells viability at 48h, with no significant difference between the two populations (Figure 5B and Supplementary Figure 5B). When seeded to form spheroids, both ALDH^high^ and ALDH^low^ 96h-old spheroids were visibly similarly sensitive to phenformin at day 10 post-treatment (Figure 5B, 5C and Supplementary Figure 5B, 5C). This was confirmed by the evaluation of total cell output/spheroid (Figure 5D and Supplementary Figure 5D) at the same timepoint, showing that phenformin leads to a comparable decrease in the overall number of cells in ALDH^high^ and ALDH^low^-derived spheroids. In our previous work, we have shown that ALDH^high^ melanoma cells are resistant to paclitaxel, a drug currently employed in the treatment of advanced-stage melanoma patients [10]. Here we observed that while ALDH^high^ cells were resistant to paclitaxel (Supplementary Figure 4E), phenformin was effective in targeting both ALDH^high^ and ALDH^low^ melanoma cells. We then wanted to confirm these results by assessing the effect of phenformin in a cellular model of melanoma that overexpresses the widely known stem cell marker SOX2. SOX2 is a transcription factor essential for maintaining the tumorigenic ability of CSC in different tumor types [49–51]. In melanoma, it promotes survival and self-renewal of CSC expressing high levels of ALDH [49]. qPCR (Figure 6A) and western blotting (Supplementary Figure 6A) analyses show that phenformin induces a significant down-regulation of SOX2 in both A375 and SK-MEL-28 cells. To evaluate whether forced expression of SOX2 could protect from phenformin cytotoxic effects, we transfected A375 cells with a SOX2-expressing vector or with an empty vector as control. Single clones were generated by antibiotic selection; SOX2 overexpression was confirmed by western blotting (Figure 6B) and immuno-fluorescence (Figure 6C and Supplementary Figure 6B). One representative A375-SOX2 clone (in which 100% cells overexpressed SOX2, Figure 6C) and one A375-CTRL clone were used. Phenformin strongly reduced the number of viable cells at 72h without any difference between SOX2-overexpressing and CTRL clone (Figure 6D). This was confirmed by viability assays, MTT and trypan blue cell counting, as shown in Figure 6E and 6F respectively. Interestingly, when we assessed the levels of ectopic SOX2 in SOX2-overexpressing cells treated with phenformin, we observed a strong decrease of SOX2 levels. This observation indicates that phenformin inhibits SOX2 expression through mechanisms that affect both its mRNA and protein.

## DISCUSSION

This study shows that modulation of melanoma cells energetic metabolism by the OXPHOS-inhibitor phenformin reduces melanoma cell viability, growth and invasion both in monolayer cell cultures and in multi-cellular spheroids. We show for the first time that phenformin reduces stem cell features in melanoma by downregulating ALDH and SOX2 expression levels. Phenformin suppresses both CSC and non-CSC cell viability and growth. By contrast metformin, a phenformin analogue, is less effective in reducing melanoma cell viability and fails to decrease cell invasion.

Using both 2D- and 3D-models we observed some discordant results in terms of drug responses [52, 53, 26], possibly due to different metabolic environments characterizing the two models. Contrarily to what observed in 2D-cell cultures, the metabolic activity of tumor cells is not homogeneous in spheroids and differs in the inner (glycolytic) vs outer (OXPHOS-dependent) layers of the spheroid structure. Since 3D cell culture approaches more accurately recapitulate solid tumor architecture, heterogeneity, differentiation and metabolism, the choice to use both 2D and 3D models in this study is of note and helps to overcome some methodological limitations of conventional 2D cell culture.

Phenformin, which is 50 fold more potent [54] and displays greater tissue bioavailability than metformin [55], markedly decreased melanoma cell viability by inducing apoptosis. Further, it reduced cell growth and invasion in both 2D and 3D models. This fits with what observed on tumor growth by Yuan and colleagues *in vivo* [40]. Interestingly, we also observed marked cytotoxic effects induced by phenformin on melanoma cells at doses lower than 1mM. Since high doses phenformin are toxic *in vivo*, our data as well as observations from other groups support its use in the clinic at lower, more tolerable doses, possibly in therapeutic combination regimens [27, 40, 51]. In addition, we observed for the first time a strong effect of phenformin on melanoma cell invasion thus indicating a potential phenformin anti-metastatic effect. Of note, phenformin activity on slow cycling BTC#2 cells is stronger as compared to fast growing cell lines. This is in line with the evidence that slow cycling, OXPHOS-dependent cells are more sensitive to drugs able to disrupt the mitochondrial respiration chain [26]. Consistently, phenformin seems to target slow cycling, JARID1B-high, melanoma cells [40].

In melanoma, MITF is both a pro-survival and a differentiating factor [57]. Interestingly, MITF drives metabolic reprogramming of melanoma cells towards mitochondrial metabolism by promoting the transcription of several genes involved in OXPHOS [58]. In line with this evidence, we observed that phenformin treatment strongly repressed MITF endogenous expression in A375 cells. This observation seems to indicate that phenformin has a transcriptional effect on MITF expression and suggests that MITF repression may be functional to phenformin activity in melanoma. However, when we tested the effect of phenformin in MITF-overexpressing melanoma cells, we did not observe any difference in drug response in MITF- and CTRL-cells. This seems to indicate that MITF is not sufficient to rescue phenformin cytotoxic effects in melanoma.

Melanoma is highly resistant to conventional treatments: even recently developed drugs show some degree of recurrence, this being mainly due to CSC persistence. Given melanoma heterogeneity and melanoma cell plasticity, the identification of a unique marker able to select cells with CSC features has been challenging [59]. We have previously shown that ALDH^high^ cells in melanoma retain a CSC phenotype and recently, a role for SOX2 in sustaining the tumorigenic ability of ALDH^high^ melanoma cells has been demonstrated [49]. Since SOX2 overexpression increases the self renewal ability of melanoma cells thus generating stem-like cells, we used this model as well as sorted ALDH^high/low^ cells to study the effect of phenformin on the stem cell compartment in melanoma. We herein show for the first time that phenformin is among the few anti-cancer drugs able to target cells with the ALDH^high^ CSC phenotype, although not specifically. Interestingly, the strong activity of phenformin on melanoma spheroids, which in turn display a higher ALDH activity as compared to monolayer cell cultures [60] (Figure 4D), seems to suggest that phenformin may have a preferential stronger cytotoxic effect on melanoma CSC. However, although ALDH^high^ cells are resistant to paclitaxel, their sensitivity to phenformin is comparable with that of ALDH^low^ counterpart. This evidence is likely due to still unraveled molecular mechanisms activated by phenformin that might be shared by ALDH^high^ and ALDH^low^ cells. Although we did not analyze glucose metabolism in melanoma CSC, it is reported that CSC often overexpress genes associated with glucose uptake, OXPHOS and fatty acid beta-oxidation [61], explaining, at least in part, the sensitivity of CSC to OXPHOS inhibitors.

Modulation of cell metabolism by inhibition of mitochondrial complex-1 has shown promising results [62]. In addition, resistance to single and combinatorial therapies is often associated with upregulation of OXPHOS [16]. Given the ability of the OXPHOS-inhibitor phenformin to target both CSC and non-CSC, our data provide evidence that phenformin might be a successful therapeutic option in melanoma, possibly in combination regimens.

## MATERIALS AND METHODS

### Reagents

Metformin (1,1-dimethylbiguanide hydrochloride), phenformin (N-(2-Phenylethyl)imidodicarbonimidic diamide monohydrochloride) and 3-(4,5-dimethylthiazol- 2-yl)-2,5-diphenyltetrazolium bromide were purchased from Sigma–Aldrich (St Louis, MO). Lipofectamine 2000 was purchased from Invitrogen (Eugene, OR). Paclitaxel was purchased from Accord Healthcare Italia.

### Cell cultures

Human B-RAF^V600E^ mutant melanoma cell line A375 was purchased from ATCC. SK-MEL-28 were a kind gift from Prof. Pincelli at the University of Modena and Reggio Emilia, Italy. Cell lines were authenticated by short tandem repeat DNA profiling after purchase. BTC#2 is a short-term melanoma cell line derived from a B-RAF^VE600E^ mutated primary melanoma patient enrolled at Arcispedale S. Maria Nuova after signing an informed consent. Once isolated, BTC#2 cells were maintained in RPMI medium supplemented with 10% (vol/vol) heat inactivated fetal bovine serum (FBS). Expression of MART-1 by immuno-cytochemistry was used to confirm the identity of melanoma cells which were 98-100% positive. A375 and SK-MEL-28 cells were cultured in DMEM medium supplemented with 10% (vol/vol) FBS or BME medium supplemented with 10% (vol/vol) FBS containing penicillin/streptomycin, sodium pyruvate (1 mM), sodium bicarbonate (1.5 g/L) and NEAA (0.1mM), respectively. All cell lines were incubated at 37°C under 5% CO2. For SOX2 and MITF stable clones derivation see supplementary M&M.

### Spheroid formation

We cultured melanoma cells either on flat bottom plates (2D-cell culture models) or round bottom ultra-low attach plates (3D-cell culture models/spheroids) in complete cell culture media. Culturing cells in ultra-low attachment plates permits growth of multi-cellular tumor spheroids that are organotypic models of solid tumor tissues. Melanoma cells were harvested from monolayer cultures, counted and resuspended into complete medium. 3000-4000 cells were seeded into each well of 96-well Corning Ultra-Low Attachment Plates (Thermo Fisher Scientific Inc., Waltham, MA) for 96h. Once formed, spheroids were incubated with vehicle or metformin/phenformin at the indicated doses up to 10 days. The stimuli were renewed every 3 days. At each timepoint, spheroids were harvested and single cell suspension was obtained by mechanical disaggregation procedure for further analyses.

### Aldefluor assay and flow cytometry

The Aldefluor kit (Stem Cell Technologies, Vancouver, Canada) was used to isolate or quantify cells with high ALDH activity. For the isolation of ALDH^high/low^ populations in melanoma, only SK-MEL-28 cells were used. For the quantification of ALDH activity in monolayer vs 3D cell cultures, A375, SK-MEL-28 and BTC#2-derived spheroids and monolayer cell cultures were analyzed at the indicated timepoints. Details on the sorting strategy and protocol are reported in the supplementary M&M section.

### 3D spheroid BME cell invasion assay

3D Spheroid BME Cell Invasion Assay was performed on SK-MEL-28 and BTC#2-derived spheroids, following manufacturer instructions (Trevigen 3-D spheroid Cell Invasion Assay, Trevigen, Gaithersburg, MD USA). We evaluated the ability of biguanides to inhibit spheroid invasion by drug-treating spheroids in invasion-permissive conditions (method I), or by pre-treating spheroids before embedding them into invasion-matrix (method II). The two methods are described in detail in the supplementary M&M section.

### Immuno-fluorescence

A375-SOX2 and CTRL clones were grown on chambers-slides for 48 hours, then washed in PBS and fixed *in situ* with buffered para-formaldehyde (4%) for 20 minutes at RT. Cells were permeabilized with 0.1% Triton X-100 for 5 minutes on ice, incubated with 2% bovine serum albumin/10% goat serum for 20 minutes, then for 60 minutes at 37°C with the mouse monoclonal anti-flag antibody (1:200, Thermo Scientific) or with FBS buffer alone as control. After three washes in PBS, cells were incubated for 60 minutes with the FITC-conjugated anti-mouse secondary antibody (Invitrogen) at 1:1000 dilution. Sections were then counterstained with Dapi (1:1000 diluted in PBS) for 5 minutes at room temperature and coverslipped with SlowFade® (Invitrogen) reagent.

### Statistical analyses

Statistical analysis was performed by Student's t-test. One or two asterisks indicate a significant difference, * 0.01 < *P* < 0.05 and ** *P* < 0.01, respectively.

## SUPPLEMENTARY MATERIALS AND METHODS AND FIGURES


