# Supplementary material for: Therapeutic potential of the metabolic modulator phenformin in targeting the stem cell compartment in melanoma
Source: Oncotarget. 2016 Dec 28;8(4):6914–28. doi: 10.18632/oncotarget.14321 (PMC5351679; doi:10.18632/oncotarget.14321)
Supplement: Supplementary file 1 [file oncotarget-08-6914-s001.pdf]

# Therapeutic potential of the metabolic modulator phenformin in targeting the stem cell compartment in melanoma

## SUPPLEMENTARY DATA

### SOX2 and MITF clones isolation

For stable clone derivation, SOX2 cDNA was cloned into the p3XFlagCMV10 (Sigma Aldrich, Milan, Italy) vector, while MITF cDNA was cloned into the pCDNA3.1 (ThermoFisher, Milan, Italy) plasmid as previously described (Sancisi et al., 2012). A375 melanoma cells were transfected either with SOX2-p3XFlagCMV10, or with MITF-pCDNA 3.1 or with pCDNA 3.1 (empty vector) using Lipofectamine 2000 (ThermoFisher) according to the manufacturer's instructions. 24H after transfection, cells were detached and seeded at clonal density in presence of 800 μg/ml Geneticin (Invitrogen Paisley, Scotland, UK). After 2 weeks of selection, single clones were picked and expanded. 2 MITF-overexpressing clones, 1 SOX2-overexpressing clone and 3 control clones (2 for MITF plasmid and 1 for SOX2 plasmid) were isolated and characterized for MITF and SOX2 expression by Western blotting and qRT-PCR.

### Western blotting

Melanoma cells were treated either with 0.5-1mM phenformin or vehicle for the indicated timepoints. Cells were lysed in Ripa buffer. Lysates were then centrifuged at 13,000 rpm at 4°C for 15 minutes to remove any cell debris. Protein concentrations were determined using Bradford assay (Biorad Laboratories Inc., Hercules CA) according to the manufacturer's protocol. Proteins were then boiled for 5 min, separated by SDS-PAGE with 4-20% gradient gels (Bio-Rad Laboratories inc., Hercules, CA) and transferred to nitrocellulose blotting membranes (Amersham Biosciences, Little Chalfont Buckinghamshire, UK). After an incubation in blocking buffer (5% Non-fat dry milk in phosphate buffered saline (PBS/0.1% Tween), the membranes were incubated with primary antibodies at 4°C. For western blotting analysis, the following antibodies were used: anti-AMPK (Cell Signalling, Hitchen, UK), anti-ALDH1A3 (Abgent, San Diego, CA), anti-MITF (Abcam, Cambridge, UK), anti-Flag (Sigma-Aldrich, St. Louis, MO), anti-SOX-2 (Cell Signalling). β-Actin (Sigma-Aldrich, St Louis, MO) served as a loading control. Bound antibody was detected using anti-mouse or anti-rabbit horseradish peroxidase-conjugated antibody and chemiluminescence (ECL Plus Kit, Amersham Biosciences, Little Chalfont Buckinghamshire, UK).

### Real time polymerase chain reaction

Total cellular RNA was extracted from sorted populations using TRI Reagent method (Sigma-Aldrich, St Louis, MO). After quantification and retro-transcription of 500 ng of total RNA (iScript cDNA kit, Biorad, Segrate, Italy), quantitative real time polymerase chain reaction (PCR) was performed with a CFX96 Real Time PCR Detection System (BioRad, Segrate, Italy) as described (Sancisi et al., 2013). Relative expression of target genes was calculated using the ΔΔCt method according to MIQE guidelines [63]. Primers for Real time PCR analysis are the following: ALDH1A3 (F:5′-ACTGCTCTCCACGTGGCATCTTTA-3′ R:5′-TGCCAACCTCTGTTGATCCTGTGA-3′), ALDH1A1 (F:5′ ACTGCTCTCCACGTGGCATCTTTA-3′ R:5′-TGCCAACCTCTGTTGATCCTGTGA-3′), MITF (F:5′ CCGTCTCTCACTGGATTGGT-3′ R:5′-TACTTGGTGGGGTTTTCGAG-3′), SOX2 (F:5′-AGC TACAGCATGATGCAGGA-3′ R:5′-GGTCATGGAG TTGTACTGCA-3′), SOX10 (F:5′-GAAGCTCGC GGACCAGTA-3′ R:5′-CGCTTGTCACTTTCGTTCA G-3′), CD271 F:5′AACCTCATCCCTGTCTATTG-3′ R:5′-GTTGGCTCCTTGCTTGTT-3′), cMET (F:5′-GG ACATCAGAGGGTCGCTTC-3′ R.5′-GGAGACACTG GATGGGAGTC-3′), Bcl-2 (F:5′-CGGAGGATGAGTG ACGAGTT-3′ R:5′-GATGTGGAGCGAAGGTCACT-3′), PGC1a (F:5′-CTGCTAGCAAGTTTGCCTCA-3′ R:5′-AGTGGTGCAGTGACCAATCA-3′). We performed three independent experiments, each one being analyzed in triplicate. A double-sided Student’s t test was performed between samples and calibrator.

### Cell viability assays

Cell viability in 2D-cell culture models was measured by MTT (3-(4,5-dimethylthiazol- 2-yl)-2,5-diphenyltetrazolium bromide) and trypan blue (TB) dye exclusion assays, as previously reported. For MTT assay, melanoma cells were seeded into each well of 96-well plates (5,000 cell/well) and treated the next day with vehicle control or biguanides (metformin 0.1, 1, 10mM; phenformin 0.1, 0.5, 1mM) for given incubation time. Viable cells were detected by incubating with MTT (Sigma-Aldrich, St. Louis, MO) solution at 37°C for 4 h. The formazan dye produced by viable cells was solubilized with DMSO and measured by a multiwell

scanning spectrophotometer at 540 nm. Results were expressed as mean±SD (n =3). Student's t-test was performed for comparison of the means. TB assay was used to count viable melanoma cells both in monolayer cell culture experiments and when melanoma cells were allowed to form spheroids. For monolayer cell cultures, cells were trypsinized at each timepoint and mixed 1:1 with trypan blue in order to count viable cells. Results were expressed as mean±SD (n =3). For 3D models, spheres were harvested at day 10 or 14, mechanically disaggregated and the number of viable cells was counted by TB dye exclusion using a hemocytometer. Results were reported as the number or % of viable/dead cells/sphere at the indicated timepoint.

## Aldefluor assay specifics

After mechanical disaggregation of spheroids, single-cell suspensions were suspended in Aldefluor assay buffer containing BODIPY-aminoacetaldehyde and incubated at 37°C for 30 minutes, as previously shown [4]. A small amount of the same cell suspension was incubated with Aldefluor buffer containing 50 mM diethylaminobenzaldehyde, an ALDH inhibitor. 7-AAD (7-Amino-actinomycin D) was used to exclude dead cells. Cell sorting and ALDH analysis were performed using a FACS-ARIA (Becton Dickinson, Franklin Lakes, NJ) and a FACS-CANTO II (Becton Dickinson), respectively. The results were analyzed using fluorescence-activated cell sorting (FACS) Diva software (Becton Dickinson). The gating strategy included the ALDHhigh gate being set at least one log apart from the $ALDH^{low}$ one. We set a cut-off of 20% $ALDH^{high/low}$ cells in order to better separate the two populations. The purity of sorted populations was analyzed and had to be greater than 95% in order to proceed with the experiments. Sorted cells were either directly lysed for expression analyses or re-seeded to perform proliferation assays.

## 3D spheroid invasion assay

In method I cells were cultured at 80% confluence, then harvested, counted and resuspended in spheroid formation matrix. This mixture was comprised of 5 µL spheroid formation ECM and 15 µL of medium with FBS and penicillin/streptomycin. Fifty microliters of cell suspension were added per well to the 3D culture qualified 96-well spheroid formation plate and centrifuged at 200 g for 3 min at room temperature and then incubated at 37°C for 72 h to promote spheroid formation. Fifty microliters of the invasion matrix was added to each well in 3D culture 96-well spheroid formation plates. The spheroid formation plate was centrifuged at 300 g at 4°C for 5 min, then transferred to the incubator at 37°C for one hour to promote gel formation. After one hour, 100 µL of complete medium containing vehicle or the indicated doses of metformin and phenformin was added to each well. The spheroid formation plate was incubated at 37°C for 1 to 5 days, and spheroids were photographed in each well every day. Using method II, we analyzed the ability of melanoma cells resistant to metformin or phenformin to preserve invasive capacity. We first generated spheroids structures as described above, then treated them with metformin or phenformin in complete medium for 72h. After pre-treatment, fifty microliters of the invasion matrix was added to each well in the 3D culture-plate. After centrifugation and gel formation, we added complete medium (without biguanides) to each well for 1 to 5 days. For both methods, a set of spheroids seeded without invasion matrix (no-matrix) was prepared in order to calculate spheroids invasion area, which is the quantification of spindle-like projections of the cells in spheroids in presence of invasion matrix. This number is calculated by subtracting the invasion area of no-matrix-spheroids to that of spheroids seeded with invasion matrix. In order to measure the viability of cells forming spheroids at the chosen timepoints, we enzymatically digested invasion matrix with 200U/ml dispase for 30 minutes and counted the number of viable cells/sphere by trypan blue cell counting. Results are expressed as the area of invasion over viable cells.

## Apoptosis analysis

Apoptosis in SK-MEL-28, A375 and BTC#2 cells was assessed using Annexin V Apoptosis detection kit (BD BD Biosciences Pharmigen, San Diego, CA). Briefly, cells were washed twice with cold PBS and resuspended with 1X Binding Buffer at concentration of 1 X $10^6$ cells/ml. 100 microliters of the solution (1 X $10^5$ cells) were incubated with 5 microliters of PE Annexin V and 5 microliters of 7-AAD. Then, cells were gently vortexed and incubated for 15 minutes at room temperature in the dark. 400 microliters of 1X Binding Buffer were added and fluorescence was measured by flow cytometry (Becton Dickinson) and analyzed by FACS DIVA software. Apotoic cell death was determined by counting the cells that stained positive for Annexin V. Apotosis was also confirmed by staining the same cells with an hypotonic solution containing 50micrograms/ml propidium iodide, 0.1% sodium citrate and 0.5% tryton X-100. After 15 minutes at 4°C in the dark cells were analysed by flow cytometry. Apoptosis was detected by evaluating the reduced fluorescence of the DNA-binding dye PI in the apoptotic nuclei.

## CD271-expression analysis by FACS

Sorted $ALDH^{high}$ and $ALDH^{low}$ SK-MEL-28 cells were immediately incubated with APC-conjugated anti-

CD271 antibody (Biolegend, San Diego CA, USA) 1:20 in PBS/BSA or with Mouse IgG1 APC-conjugated Antibody (isotype control), for 30 min at 4°C. Then, they were washed with PBS/BSA and stained with 7-AAD for 10 minutes at 4°C in order to eliminate dead cells from the analysis. Cells were analyzed using an FACS-ARIA flow cytometer (Beckman Coulter).

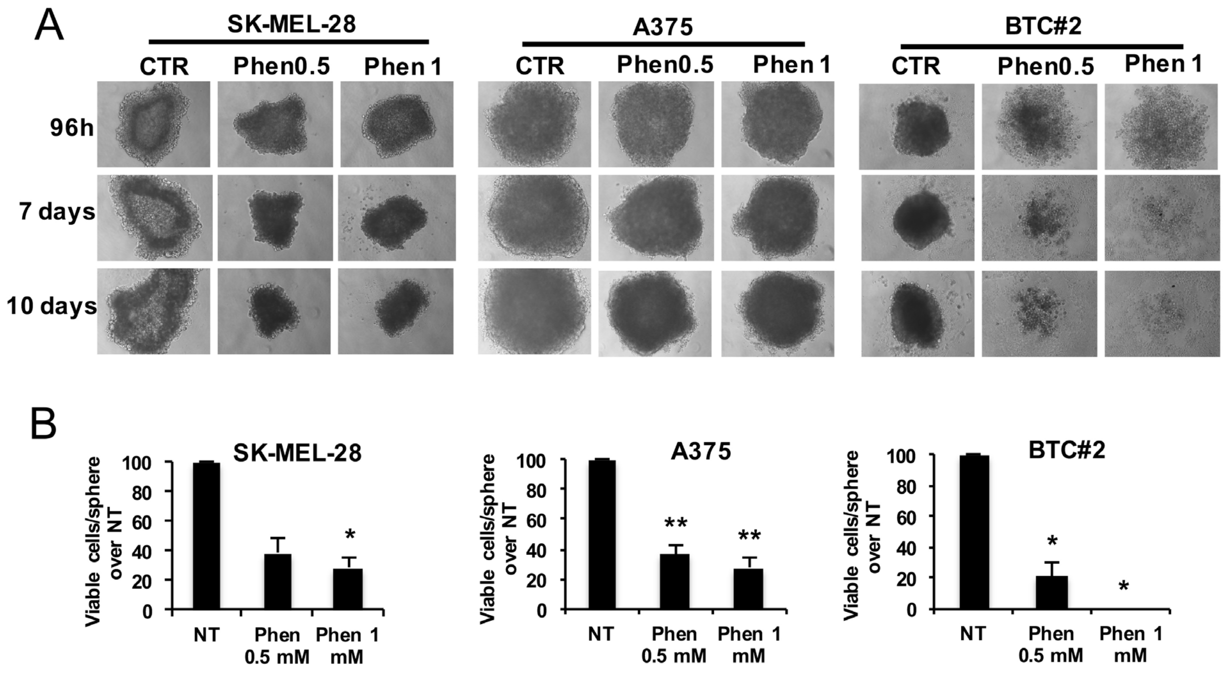


**Supplementary Figure 1: A.** Melanoma cells were seeded in ultralow-attachment plates in complete medium for 72h. Once formed, spheroids were treated with 0.5-1mM phenformin and photographed at indicated timepoints. **B.** At day 10, spheroids were harvested, mechanically disaggregated and viable cells were counted by trypan blue staining. Data represent the mean ±SD of the experiment performed in triplicate and are represented as the % of viable cells/spheroid over untreated (NT) spheroids. Student T-test was performed for statistical analysis of data (* $p<0.05$; ** $p<0.01$).

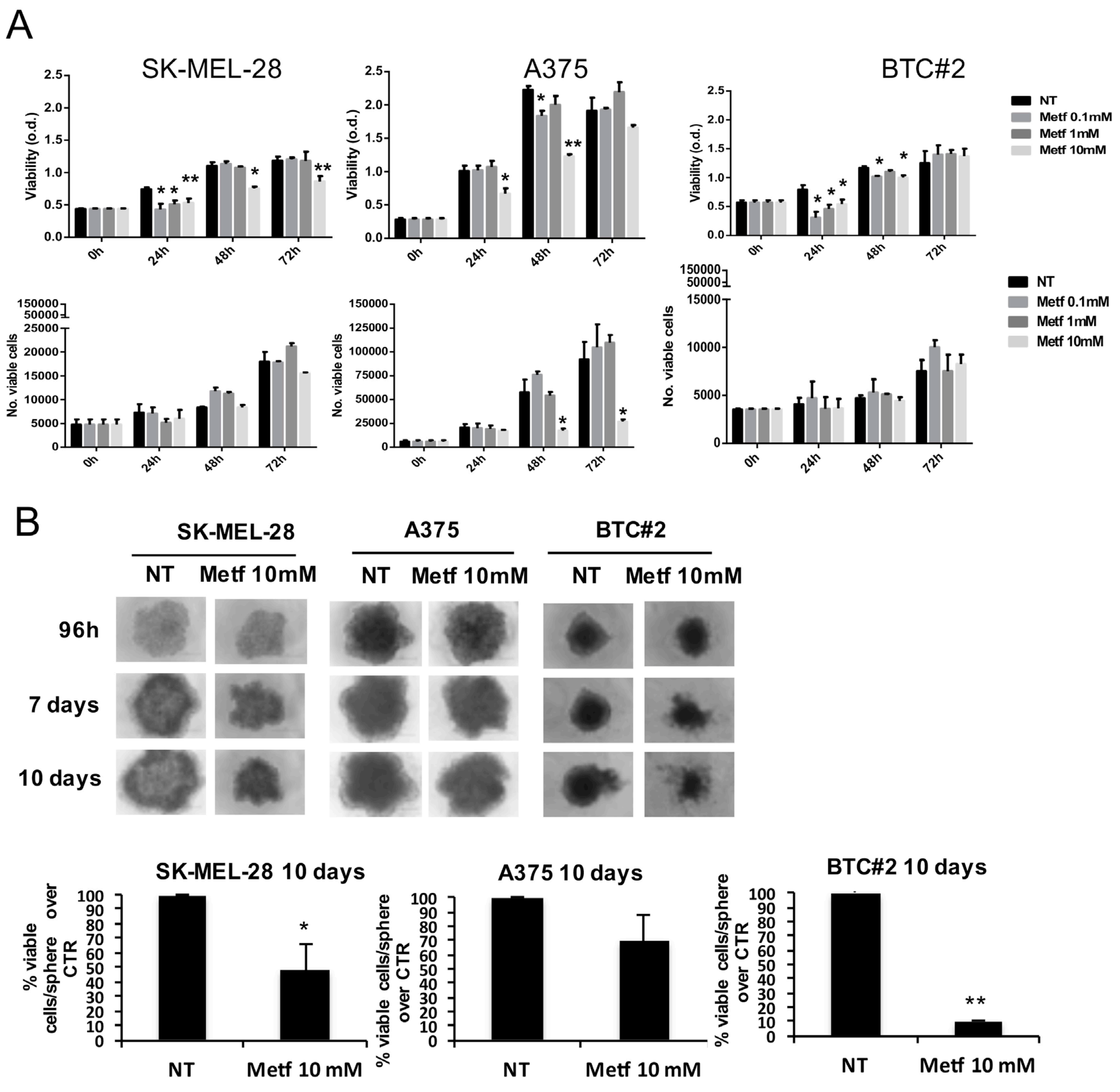


**Supplementary Figure 2: A.** Melanoma cells were seeded, treated with 0.1-10mM metformin and MTT assay (upper panel) or blue trypan cell counting (lower panel) were performed up to 72h after treatment. **B.** Melanoma cells were seeded in ultralow-attachment plates in complete medium for 72h. Once formed, spheroids were treated with 10mM metformin and photographed at indicated timepoints (upper panel). At day 10, spheroids were harvested, mechanically disaggregated and viable cells were counted by trypan blue staining. Data represent the mean ±SD of the experiment performed in triplicate and are represented as the % of viable cells/spheroid over untreated (NT) spheroids (lower panel). Student T-test was performed for statistical analysis of data (* $p<0.05$; ** $p<0.01$).

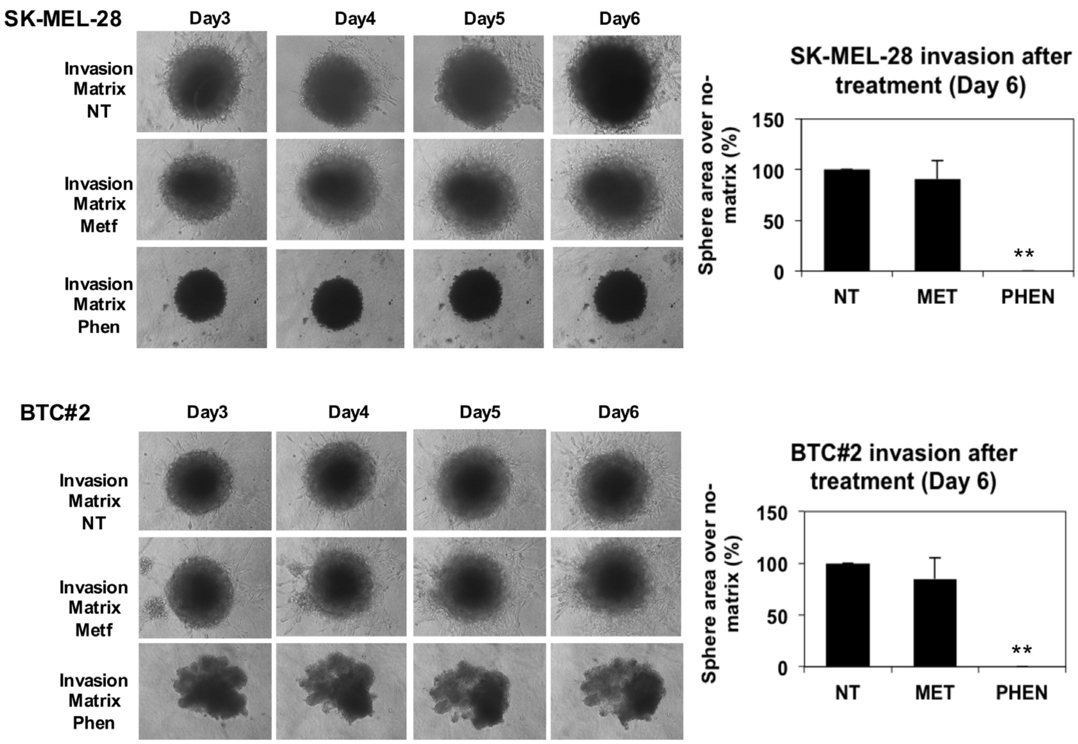


**Supplementary Figure 3: Melanoma cells were resuspended in spheroid formation ECM, seeded in a 3D culture qualified 96-well spheroid and maintained up to 72 h to promote spheroid formation.** Then, spheroids were treated either with vehicle, 10mM metformin or 1mM phenformin. After 72h, invasion matrix and complete medium were added to the wells. Pictures of spheroids were taken up to day6. Invasion area at day6 was calculated by using ImageJ software. Bar graphs display the area of invasion of NT or treated spheroids over no matrix spheroids and are expressed as % of invasion over NT. Error bars represent mean ±SD of three independent experiments. Student T-test was performed for statistical analysis of data (* $p<0.05$; ** $p<0.01$).

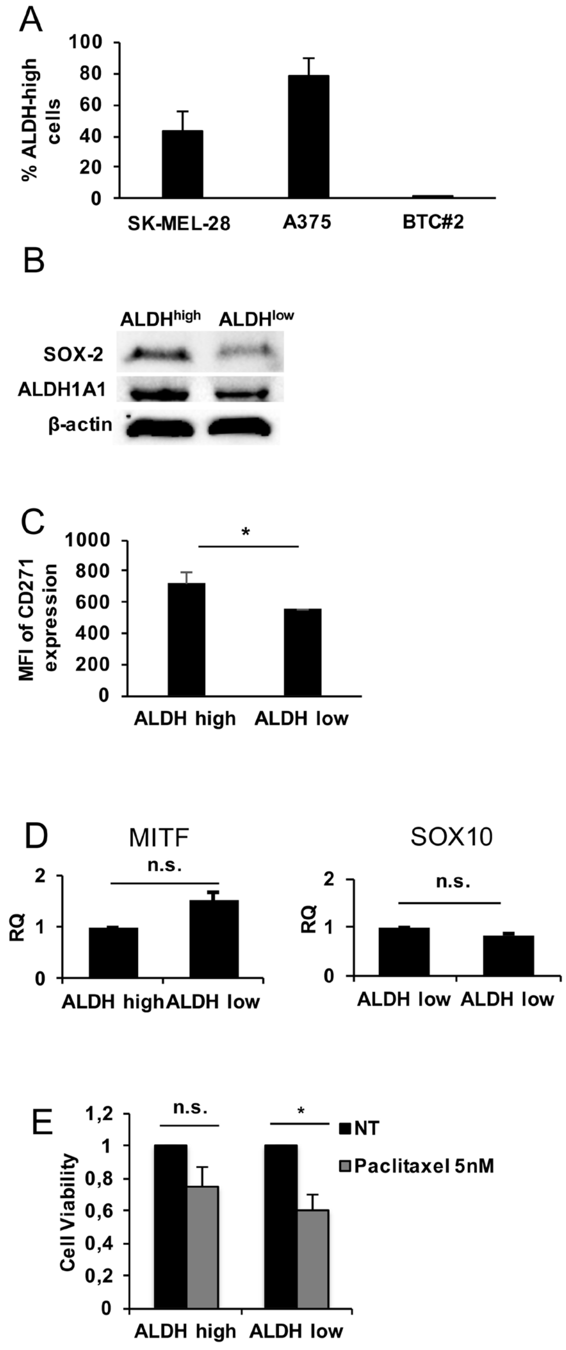


**Supplementary Figure 4: A.** A375, SK-MEL-28 and BTC#2 cell lines were seeded at the same plating density and harvested 24h later for Aldefluor analysis by FACS. The % of ALDH$^{high}$ cells was shown; error bars represent mean ±SD of three independent experiments. **B.** Sorted ALDH$^{high}$ and ALDH$^{low}$ SK-MEL-28 cells were immediately lysed for SOX2 and ALDH1A1 protein expression by western blotting. Western blottings are representative of an experiment performed in triplicate (three biological replicates). β-actin was used as loading control. **C.** Sorted ALDH$^{high}$ and ALDH$^{low}$ SK-MEL-28 cells were immediately stained with APC-conjugated anti-CD271 antibody or Mouse IgG1 APC-conjugated antibody and analysed by flow cytometry. Mean Fluorescence Intensity (MFI) of CD271 expression is shown. Student T-test was performed for statistical analysis of data (* $p<0.05$; n.s. not significative). **D.** Sorted ALDH$^{high}$ and ALDH$^{low}$ SK-MEL-28 cells were immediately lysed for mRNA analysis by real-time PCR for the expression of MITF and SOX10. Data are the mean ±SD of the experiment performed in triplicate. Student T-test was performed for statistical analysis of data (* $p<0.05$; ** $p<0.01$). **E.** Sorted ALDH$^{high}$ and ALDH$^{low}$ SK-MEL-28 cells were treated with or without 5nM paclitaxel and cell viability was measured by trypan blue cell count at 48h. Error bars represent mean ±SD of three independent experiments. Student T-test was performed for statistical analysis of data (* $p<0.05$; n.s. not significative).

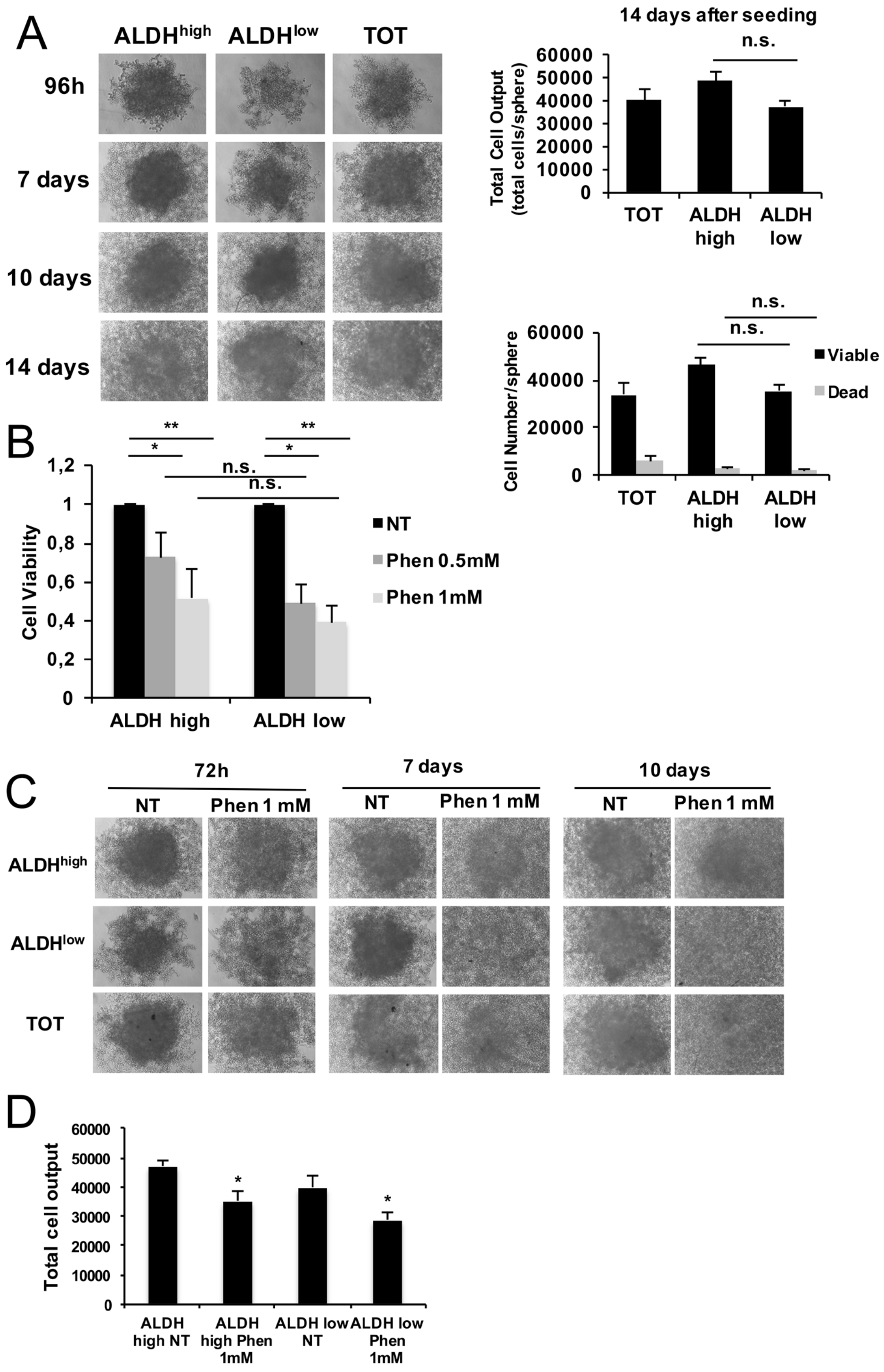


**Supplementary Figure 5: A.** Sorted ALDH$^{high}$ and ALDH$^{low}$ A375 cells were seeded in complete medium in order to form spheroids and photographed at different timepoints. At day 14, spheroids were mechanically disaggregated and viable cells were counted by trypan blue. Bar graphs display total cell outputs (viable+dead cells) per spheroid (right, upper graph) or the cell number/sphere (right, lower graph). **B.** Sorted ALDH$^{high}$ and ALDH$^{low}$ A375 cells were seeded in complete medium on 96-wells plates and treated with 0.5-1 mM phenformin. Cell viability at 48h was measured by trypan blue cell count. **C.** Sorted ALDH$^{high}$ and ALDH$^{low}$ SK-MEL-28 cells were seeded in complete medium on ultralow-attachment plates to form spheroids, then treated with 1mM phenformin up to 10 days. Photographs of treated spheroids were taken at different timepoints. **D.** At day 10, spheroids were mechanically disaggregated and viable and dead cells were counted by trypan blue. Bar graphs display total cell outputs (viable+dead cells) per spheroid. Error bars represent mean ±SD of three independent experiments. Student T-test was performed for statistical analysis of data (* $p<0.05$; ** $p<0.01$; n.s. not significative).

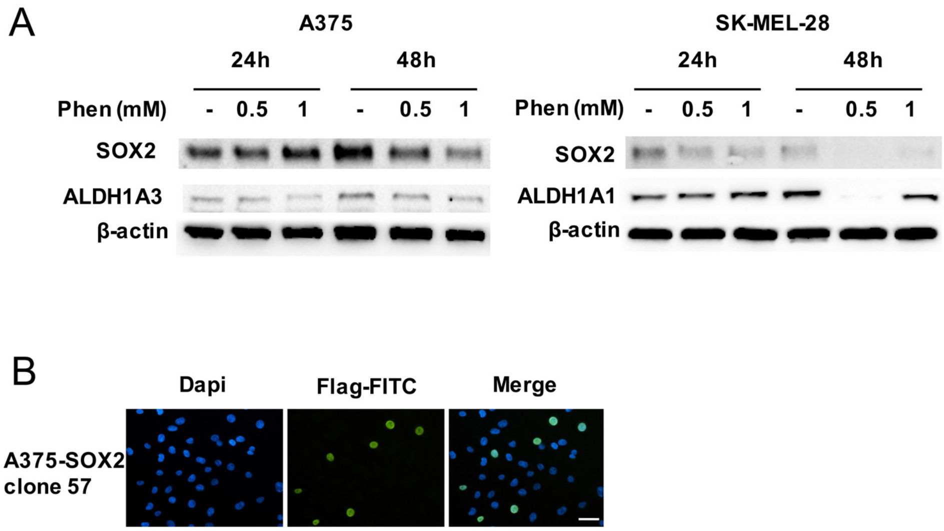


**Supplementary Figure 6: A.** Total cell lysates of SK-MEL-28 and A375 melanoma cells treated with or without 0.5-1mM phenformin up to 48h were immunoblotted with anti-human SOX2 and ALDH1A3 antibodies. Western blottings are representative of an experiment performed in triplicate (three biological replicates). β-actin was used as loading control. **B.** Immunofluorescence was performed on A375-SOX2 clone 57 by using anti-flag antibody (green) and dapi for nuclear staining (blue). The selected clone contains 13% flag-positive cells and was therefore not included in the study (scale bar: 25µm).
